# Advanced gustometer design for reliable recording of gustatory event-related potentials in healthy young adults

**DOI:** 10.3389/fnins.2025.1534296

**Published:** 2025-03-26

**Authors:** Zhongyan Chen, Xiaolin Zhou, Chunjing Li, Jianfeng Liu

**Affiliations:** ^1^Department of Otorhinolaryngology Head and Neck Surgery, China-Japan Friendship Hospital, Beijing, China; ^2^Department of Otorhinolaryngology Head and Neck Surgery, Beijing Changping Hospital of Integrated Chinese and Western Medicine, Beijing, China; ^3^Chinese Academy of Medical Sciences and Peking Union Medical College, Beijing, China; ^4^Beijing Yuda Medical Technology Co., Ltd., Beijing, China

**Keywords:** gustatory event-related potentials, gustometer, taste, salty stimulation, psychophysical tests

## Abstract

**Introduction:**

This study introduces an advanced gustometer to record Gustatory Event-Related Potentials (GERPs) in healthy young adults. We aimed to validate its functionality and reliability.

**Methods:**

The gustometer includes a programmable controller, a human-machine interface, a modular pump system, and supporting hardware. The Neuro-Audio EEG platform recorded EEG data from 46 volunteers. Psychophysical gustatory tests assessed gustatory function. GERPs were tested using distilled water as a control and sodium chloride solutions (0.3 and 0.6%) as tastants. Tetracaine anesthetized the tongue surface to observe waveform changes and confirm GERP specificity. GERP responses were recorded at the Fz and Cz sites, focusing on the latency and amplitude of GERP P1 and P2 waves and their correlation with psychophysical test results. No stable waveforms were recorded with distilled water.

**Results:**

All subjects displayed stable GERP waveforms following salty stimulation. These waveforms disappeared post-anesthesia, confirming GERP specificity. The recorded GERP comprised P1-N1-P2 components. The latency of P1 and P2 waves decreased with increasing salt concentration (*p* < .05). No significant differences in latency were observed between the Fz and Cz sites. Additionally, 48% of subjects showed increased P1-N1 and P2-N2 amplitudes with higher salty stimulation. The latency of P1 and P2 positively correlated with psychophysical test results.

**Discussion:**

This novel gustometer effectively evoked reliable GERP waveforms. The study validated the consistency of GERP amplitude and latency with psychophysical tests, highlighting the gustometer’s potential for clinical and research applications in gustatory system.

## 1 Introduction

Taste function not only enhances our culinary experiences and social interactions but also plays a crucial role in the detection of toxic substances. Taste dysfunction can arise from various conditions, including neurological disorders, head trauma, and infections. Estimates indicate that the prevalence of complete ageusia ranges from 0.84% to under 4% (Deems et al., 1991), while hypogeusia can affect up to 20% of individuals attending chemosensory clinics (Vennemann et al., 2008). Research by Antje Welge-Lüssen et al. suggests that approximately 5% of the general population experiences hypogeusia. However, complete ageusia is extremely rare, occurring in only one or two individuals per 1,000 (Deems et al., 1991; Welge-Lussen et al., 2011). Notably, the elderly have exhibited a significant rise in taste disorder prevalence, reaching 14–22%, which is much higher than olfactory dysfunction (Boesveldt et al., 2011). The advent of COVID-19 has underscored its role as a prominent cause of taste impairments, with studies reporting an increased prevalence of hypogeusia (12–28%) among infected individuals (Jafari et al., 2021; Imoscopi et al., 2012; Hintschich et al., 2023; Jianfeng et al., 2008). Such high prevalence, combined with its potential consequences—including poor appetite, malnutrition, depression and anxiety disorders, and compromised quality of life (Noel et al., 2017)—emphasizes the importance of gustation-monitoring and developing effective diagnostic assessments for gustatory dysfunction.

To the best of our knowledge, methods used for assessing taste disorders include: subjective methods (taste questionnaires and psychophysical methods), electrophysiological tests, blood tests, and imaging assessments (positron emission tomography, PET; functional MRI) (Nin and Tsuzuki, 2024). Although psychophysical tests are widely used for routine assessment of gustatory sensitivity in clinical practice, they may not be feasible for children, potential malingerers, demented patients, or for medico-legal purposes. Moreover, the complex formation of taste, which is an aggregate outcome of retronasal olfaction (oral cavity and nasopharynx), mechanical and chemical sensitivity through the trigeminal nerve, and the gustatory system itself, often leads to confusion with olfactory function (Fark et al., 2013). To address this, electrophysiological testing, specifically gustatory event-related potentials (GERPs) tests, have been employed (Fark et al., 2013).

Since first introduced in 1971 (Funakoshi and Kawamura, 1971), GERPs have received wide attention for their ability to objectively, reliably, easily, and inexpensively assess the early cortical response to gustatory stimuli (Hu et al., 2020). GERPs have been obtained in response to sodium chloride (salty), sugar solutions (sweet) (Hu et al., 2020; Ohla et al., 2012; Ohla et al., 2010), acetic acid (sour), bitter, and umami (Hummel et al., 2010), demonstrating the activation of the gustatory pathway from the tongue receptors to the taste cortex by taste stimuli. GERP is defined by three peaks: P1, N1, and P2. The P1 peak corresponds to the beginning of the GERP. P1 and N1 peaks are described as the sensory cerebral response (Hu et al., 2020); the P1-N1 amplitude corresponds to the intensity of the cerebral activation. The P2 peak is described as the subjective interpretation of the gustative stimulus by the subject (cognitive response) (Zhu et al., 2023). The temporal features of GERPs, such as latency and amplitude, have been found to vary with the concentration and palatability of the stimuli (Payne et al., 2024).

However, unlike the widespread adoption of EEG in clinical practice, the application of GERP remains relatively limited, primarily due to the scarcity of mature commercial systems (Hu et al., 2020; Iannilli et al., 2015; Mouillot et al., 2019; Mizoguchi et al., 2002; Jacquin-Piques et al., 2015; Andersen et al., 2019). GERPs represent an aspect of action potentials, and their recording is relatively straightforward. Therefore, the development of a gustometer has become a focal point in the research of GERPs. Firstly, taste is a chemical sense that requires stimulation through a natural chemical sensing process to obtain ideal evaluation results. Secondly, tastants should be delivered on the tongue surface without producing artifacts such as thermal, tactile, or nociceptive co-activation.

Our team has been immersed in both the basic and clinical research of chemosensation, focusing on olfaction and taste (Jianfeng et al., 2008; Noel et al., 2017). The aim of the present study was two-fold. Firstly, to present our newly established gustometer, detailing the construction of the gustatory stimulator, the functionality of the device, the taste delivery mode, and the parameters for taste stimulation. Secondly, to utilize this system to investigate the GERPs for salt taste in terms of validity and reliability in a cohort of healthy young Chinese individuals. We hope to obtain fundamental data for GERP components within the healthy Chinese population and to perform comparative analyses with other centers to confirm the feasibility, reliability, and specificity of our GERPs platform.

## 2 Methods

### 2.1 Subjects

Forty-six healthy subjects (14 men and 32 women) were enrolled in this study. All these subjects had normal taste confirmed with psychophysical gustatory test (3-droplet method). The mean age was 25.4 ± 3.6 years. All of the subjects were nonsmokers. None of the subjects had oral, dental, or neurological disorders or specific medical histories. Subjects who were currently undergoing medical treatment and obese subjects were excluded.

### 2.2 Ethical approval

The subjects were informed about the nature and aims of the experiment, and each of them provided written consent. The study was approved by the China-Japan Friendship Hospital Ethical Committee (2022-KY-080), in accordance with the latest revision of the Declaration of Helsinki.

### 2.3 Procedure

#### 2.3.1 Psychophysical gustatory test (3-droplet method)

Different concentrations of sucrose (sweet), NaCl (salty), citric acid (sour), and quinine hydrochloride (bitter) solutions were prepared. The psychophysical gustatory test was conducted using the three-droplet method, with minimum concentrations of sucrose (0.19 g/mL), NaCl (0.06 g/mL), citric acid (0.15 g/mL), and quinine hydrochloride (0.0012 g/mL) (Pingel et al., 2010). One drop of tastant was dripped onto the middle of the anterior two-thirds of the extended tongue with pipettes. The subject was asked to choose from four tastes: sour, sweet, bitter, and salty (multiple forced choice task). The score for each taste was graded from 10 to 1 with increasing concentrations and was recorded when a taste was identified twice consecutively.

#### 2.3.2 Construction of a gustometer

**Figure 1:**
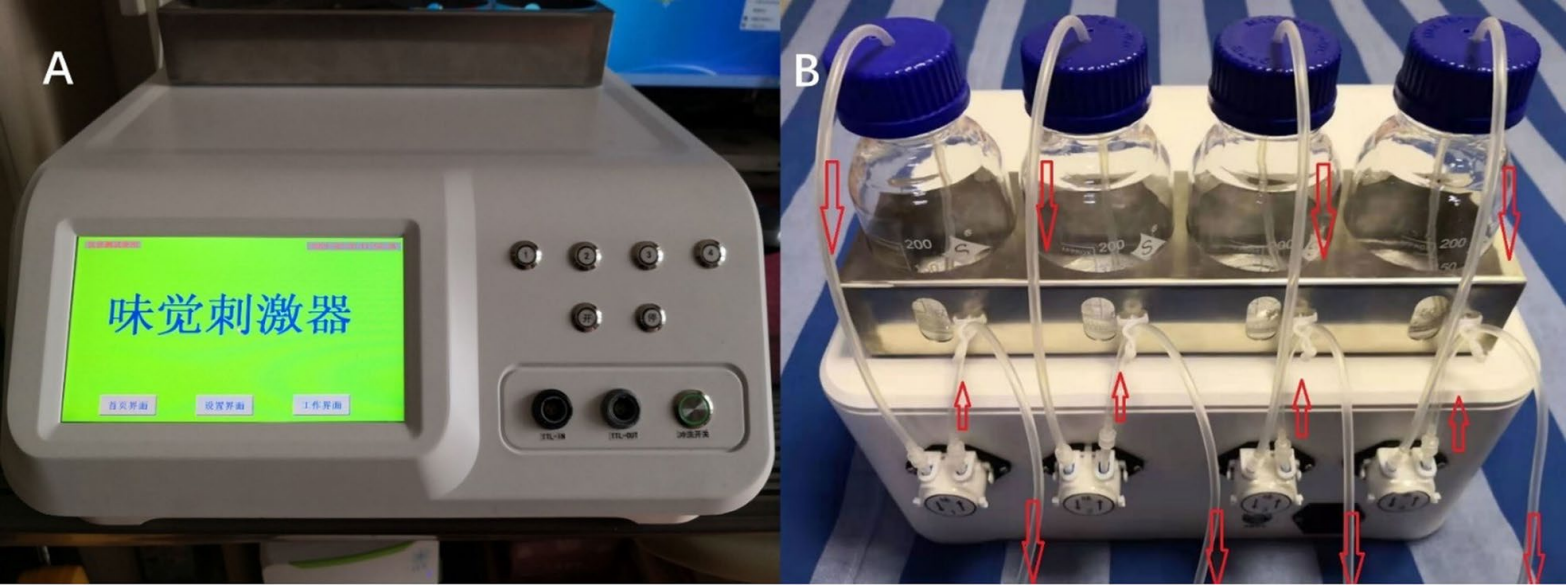
Photographs of the gustometer, showing its front **(A)** and back **(B)** views.

**Figure 2:**
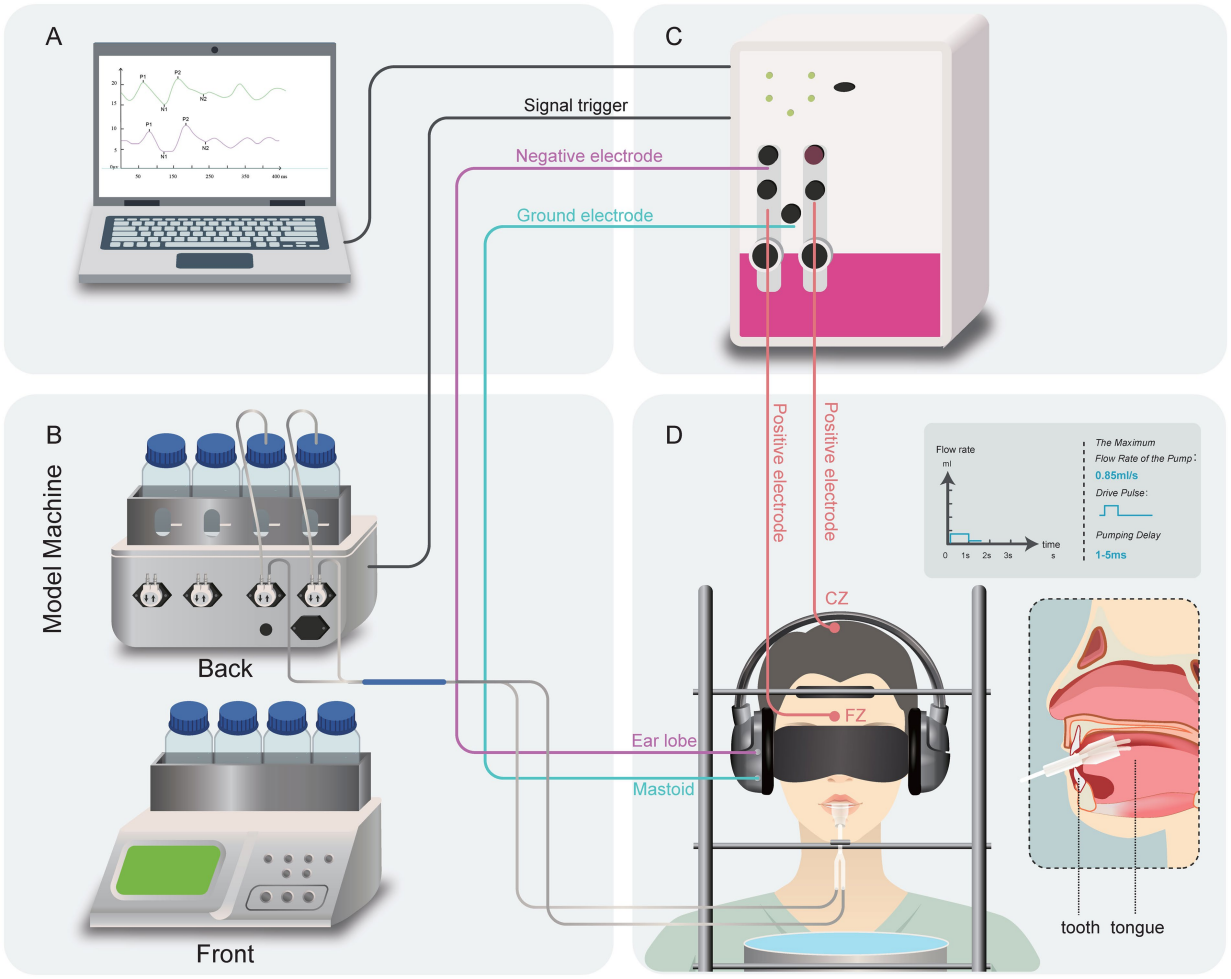
Schematic illustration of the gustometer. The front and back of the gustometer machine consists of a system of computer **(A)** controlled pumps (channel 1–3 for tastants, channel 4 for distilled water). The tastant solutions are contained in glass bottles **(B)**; GERPs was recorded via Neuro-Audio EEG system **(C)**; During testing, visual stimuli and auditory stimuli, as well as swallowing movements, were avoided. **(D)** The pump’s drive pulse is a pulsatile flush with a delay of approximately 1–5 ms, and the maximum pump flow rate is 0.85 mL/s.

The gustometer is a key component in the apparatus used for GERPs recordings, as shown in Figures 1, 2. The device consists of a main host and auxiliary parts. The main host includes a human-machine interface touchscreen, a programmable controller, a mechanical control panel, input/output interfaces, and a peristaltic pump. The touchscreen handles system interactions, parameter settings, and command execution, while the programmable controller manages pump operation to precisely dispense the test liquid. Heating and cooling modules keep the stimuli at a temperature close to that of the tongue. The auxiliary components include a liquid bottle stand, bottles, pump tubes, connectors, and a mouthpiece. The stimulator’s external output has four channels: Channels 1–3 deliver taste solutions, and Channel 4 provides distilled water.

**Figure 3:**
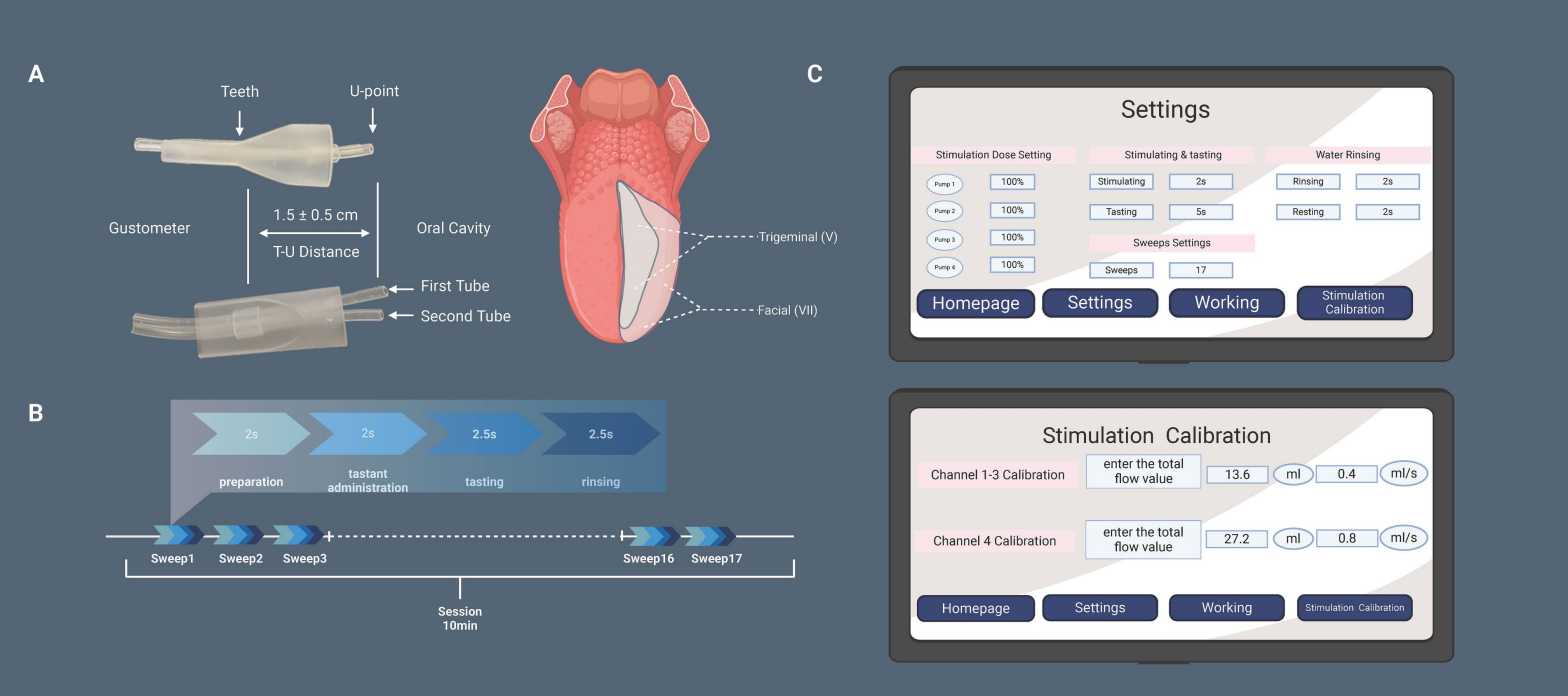
**(A)** The upper image shows a side view and the lower image a front view of the mouthpiece, indicating the position where it is held between the teeth. The distance between the teeth contact point and the U point is adjustable to 1.5 ± 0.5 cm, allowing for precise positioning of the internal Teflon tubing. This ensures that the taste solution stimulates the lateral anterior part of the tongue—innervated by the facial nerve—without activating the trigeminal nerve. **(B)** The session timeline illustrates that each stimulus consists of four phases: preparation, taste stimulus delivery, tasting, and rinsing. Each session includes a total of 17 stimuli (sweeps). **(C)** The gustometer’s human-machine interface is displayed, showing parameter settings that allow for independent adjustments of each of the four channels. The stimulation calibration screen enables adjustments to the flow rate parameters.

The stimulus parameters are configured via the human-machine interface touchscreen (Figure 3C), allowing for the selection of tastants, number of sweeps, channel flow rate calibration, and phase duration for each step of the process (including stimulation dose setting, tastant delivery, tasting, and water rinsing) within a single sweep. Once the test begins, the main host sends synchronized commands to both the mechanical control panel and the GERP recording Neuro-Audio system through a synchronization trigger line (Figure 2), with a precision of 1 ms.

Two parallel silicone tubes were used: one from channel 4 for water (Second Tube) and the other from bottles 1–3 for a taste solution (First Tube). When you need to change the taste stimulants, switch the First Tube between bottles 1–3. It is important to activate the stimulator’s flushing function before starting the actual stimulation.

For each channel, the maximum flow rate is 0.85 mL/s, with stimulation delivered as a drive pulse. The pump action delay ranges from approximately 1–5 ms, and the stimuli are square waves with a rapid rise time (Figure 2D). At the beginning of each stimulation cycle, the First Tube delivers the taste solution to the lateral side of the tongue in a pulsatile manner for 2 s, followed by a 2-s tasting period. During stimulus presentation, water supply through the second tube is temporarily suspended to maintain stimulus integrity. The rinsing phase utilizes distilled water delivered through Channel 4 at a flow rate of 0.8 mL/s for effective tongue cleansing. The flow rate of 0.4 mL/s for the taste solution was selected based on literature recommendations (Mizoguchi et al., 2002; Hummel et al., 2010).

#### 2.3.3 The design of the mouthpiece

The ends of the two silicone tubes emanating from the main unit are encased in a plastic mouthpiece, as shown in Figure 3A, which illustrates the structure of the mouthpiece from both the front and side views. This mouthpiece can be held between the teeth, with the ends of the tubes directed toward the oral cavity (Figure 2D). The tubes were positioned at a distance of 1.5 ± 0.5 cm (T-U distance) from the dental arch along the midline of the tongue. The T-U distance can be adjusted so that the U point is precisely located laterally to the anterior third of the tongue, avoiding stimulation of the trigeminal nerve area. Solutions were delivered to the tongue through the ends of each tube (U point).

When conducting GERP (Mastinu et al., 2024):

1. Subjects were asked not to eat, or drink anything but water during the time between meals and the GEP recording.
2. The recording device needs to be placed inside an electromagnetic and sound shielding chamber to eliminate potential electromagnetic interference and ensure the stability of the recorded waveforms, with the EEG and the gustometer placed in two separate spaces.
3. Participants wear noise-canceling headphones to mask switching clicks of the stimulation device and other possible sounds.
4. Participants should wear a blindfold to avoid light stimulation. Hence, no blink artifacts contaminated the recordings.
5. The subject’s chin rested on a tray, with their head tilted downward, allowing the water to flow directly into a catch tray below after stimulating the tongue via the mouthpiece, thus avoiding the effects of swallowing actions (Figure 2).
6. They were asked to count the number of stimuli in order to focus the participants’ attention.

#### 2.3.4 Taste stimuli

The stimuli were applied to the anterior tongue via the one of the tube from the mouthpiece as shown in Figure 2. Taste stimuli (the sodium chloride (NaCl) solution) was applied in two different concentrations (weak and strong: 0.3 and 0.6%, equal to 51 and 103 mM, respectively). The distilled water was used as a blank control. After the subjects’ anterior tongues were sprayed with 1% tetracaine to anesthetize the areas innervated by the trigeminal and facial nerves on the tongue surface, the waveforms were retested. To further confirm whether tetracaine-induced surface anesthesia impaired taste perception, the subjects were asked to rate the intensity of saltiness. All 10 patients reported that the salt taste was “not detectable.”

For each stimulus presentation, the process begins with the administration of a taste solution (default duration of 2 s), followed by a tasting phase (default duration of 5 s), and concludes with distilled water to rinse the tongue through channel 4 (default duration of 2 s). This constitutes a single stimulation (sweep), with a 2-s interval/rest between two sweeps (Figure 3B).

#### 2.3.5 GERP recording and data analysis

**Figure 4:**
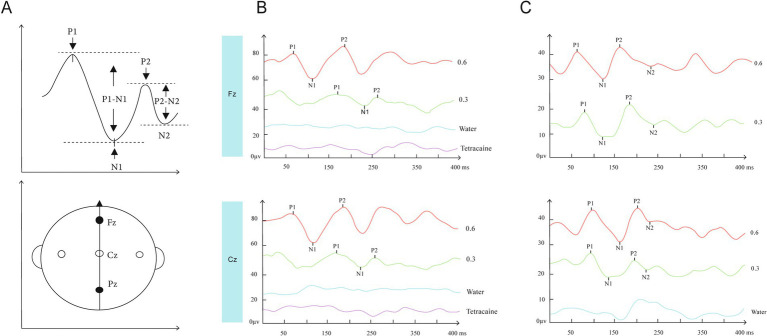
**(A)** schematic drawing of ERP peaks and EEG recording position. **(B,C)** show typical recordings from 2 subjects (No 45, 38 respectively) for all experimental conditions plus the corresponding Grand Means. GERP at recording position Fz and Cz in response to distilled water (blue), 0.3% (green) and 0.6% NaCl (red) from an individual subject. Under water stimulation, no GERPs were recorded at Fz and Cz. Compared with lower salty concentration, GERP evoked by higher salty concentration at both two recording sites had larger P1-N1 and P2-N2 amplitude **(B,C)** and shorter P1 and P2 latency. After the subjects’ anterior tongues were sprayed with 1% tetracaine to anesthetize the areas innervated by the trigeminal and facial nerves on the tongue surface, the GERPs were tested again using the NaCl stimulus, and the aforementioned waveforms disappeared (**B**, purple).

Continuous EEG was recorded and averaged via the Neuro-Audio EEG device (Neurosoft, Ivanovo, Russia). Due to the Neuro-Audio EEG device having only 2 channels, as described by a previous study (Zhu et al., 2023), central electrode Cz and frontal electrode Fz were selected for recording (Figure 4A). The electrodes were referenced against linked earlobes, and the ground electrode was placed on the mastoid. GERPs were recorded by surface electrode with impedance <5 kΩ. A signal was sent to Neuro-Audio when a taste solution was administered (with 1 ms precision), to obtain a precise time recording of GERP. GERP analysis was performed with the same software. Each subject’s data were digitally filtered using a 0.1–30 Hz bandpass filter. The sampling frequency was 512 Hz. The recorded GERP signals were averaged with 17 stimulus presentations.

GERP was defined by four peaks, as described in previous studies (Ohla et al., 2012; Ohla et al., 2010). P1, the first positive peak; N1, the first negative peak; and P2, the second positive peak. P1 latency (in ms), P2 latency (in ms), P1-N1, and P2-N2 amplitude (in μV) of the GERPs were registered for each recorded electrode and each tastant concentration. The P1 latency was defined as the time interval between stimulus delivery and the potential positive peak P1. The P2 latency was defined as the time interval between the stimulus delivery and the second positive peak. The amplitude of each response was calculated as the height between the first positive and the negative peaks (P1-N1 amplitude) (shown in Figure 4A) (Mouillot et al., 2019). The positive peak corresponded to the peak pointing down, whereas the negative peak corresponded to the peak pointing up.

### 2.4 Statistics

Continuous and categorical variables were expressed as mean ± standard or numerals (percentages), respectively. Components of gustatory ERPs from different recording sites and two concentrations were compared using *t* test. The correlation between psychophysical gustatory test scores and GERPs parameters was analyzed with simple linear regression. A *p*-value of <0.05 was considered statistically significant. Statistical analysis was performed with Prism 10 (Version 10.1.1, GraphPad Software, Boston, Massachusetts USA).

## 3 Results

### 3.1 NaCl solution as a salty tastant elicits corresponding potentials

**Figure 5:**
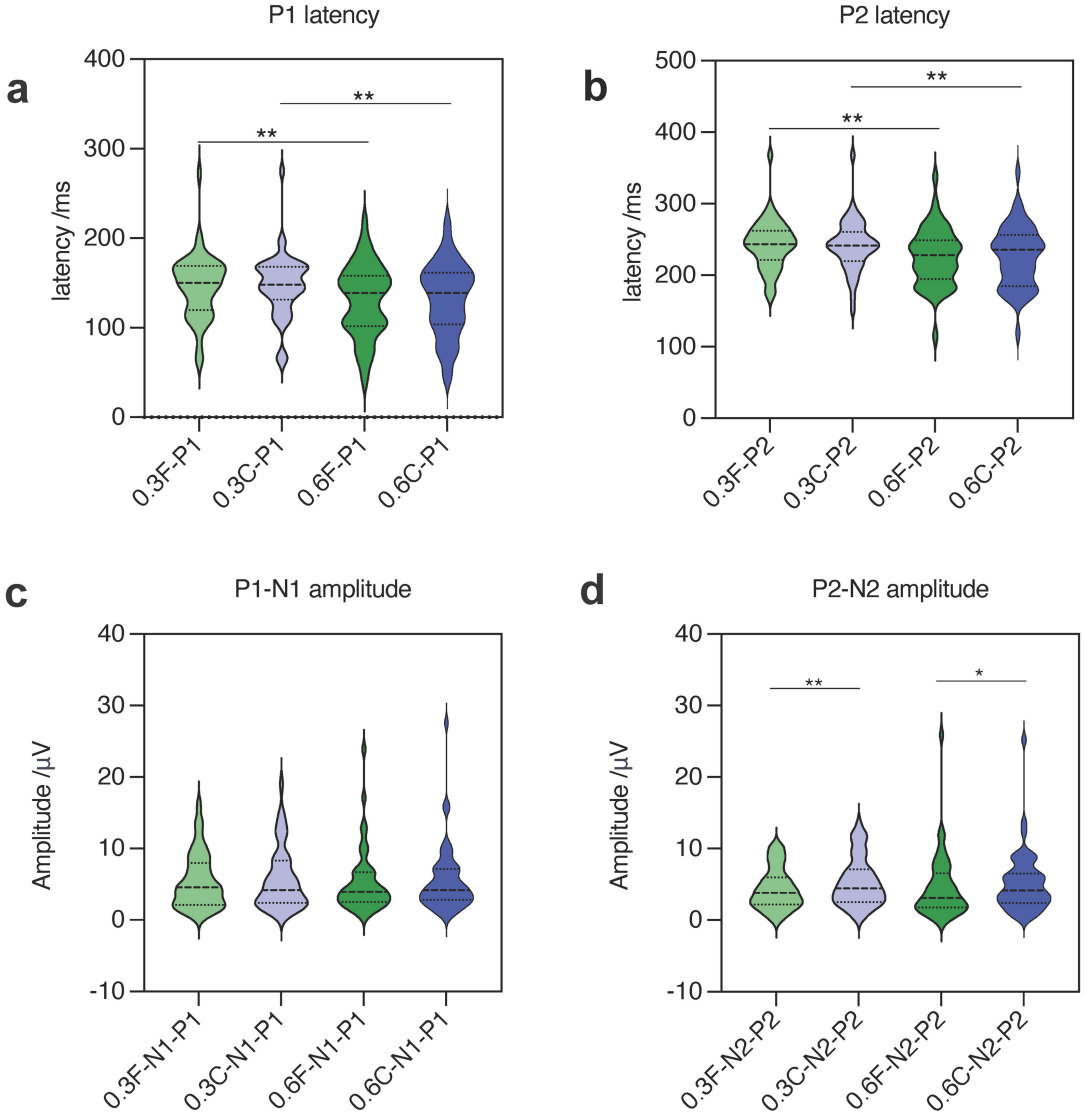
Topographical and concentration distribution of GERPs latency **(a,b)** and amplitude **(c,d)**. (F: Fz; C: Cz; 0.3: 0.3% NaCl; 0.6: 0.6% NaCl).

All subjects (*n* = 46) underwent recording and analysis at Fz and Cz sites in response to salt stimuli, and four distinct peaks were observed for all subjects. P1 is the first positive peak; N1 is the first, higher and negative peak; P2 is the second positive peak; N2 is the second negative peak (Figure 4). The latencies (ms) of P1, P2, and the amplitudes (μV) of N1-P1 and N2-P2 of GERPs were recorded. The average latencies and amplitudes of the two peaks P1 and P2 recorded at Fz and Cz at two salty concentrations are shown in Table 1. Representative recordings of GERPs on electrodes Fz and Cz from two subjects are shown in Figure 4. Significant differences between responses at the different recording sites (Fz-Cz) were observed for averaged amplitudes of P2-N2 for both salt concentrations (0.3, *p* < 0.01; 0.6 *p* < 0.05). The GERPs recorded at Cz displayed higher P2-N2 amplitude than Fz, as shown in Figure 5. No significant differences were found with regard to latencies of peaks P1 and P2 between Fz and Cz positions.

**Table 1:** Grand averaged latencies and amplitudes of GERPs at Cz and Fz evoked via two concentration NaCl solutions.

	0.3-Fz	0.6-Fz	0.3-Cz	0.6-Cz
P1 latency (ms)	146.2 ± 36.3	130.9 ± 40	147 ± 35.7	132.6 ± 41
N1 latency (ms)	191 ± 36.4	176.8 ± 40	190.7 ± 36.3	177.8 ± 41.4
P2 latency (ms)	242.9 ± 34.9	225.6 ± 40	241.6 ± 35.8	226.2 ± 43.3
N2 latency (ms)	290.3 ± 36.2	267.6 ± 44.7	290.3 ± 36.2	268.1 ± 47.8
P1N1 amplitude (μV)	5.3 ± 3.8	5.2 ± 4.4	5.6 ± 4.3	5.6 ± 4.8
P2N2 amplitude (μV)	4.4 ± 2.8	4.6 ± 4.5	5.3 ± 3.3	5.2 ± 4.3

### 3.2 No EEG waveforms recorded with distilled water and EEG by NaCl disappeared after tongue surface anesthesia

To demonstrate that the aforementioned GERPs were uniquely evoked by NaCl, this experiment also analyzed the waveforms evoked by distilled water as controls for comparison in 10 subjects (No.1–10). It was found that no significant waveforms were elicited with distilled water stimulation. Furthermore, for the same 10 subjects, after local surface anesthesia of the tongue with 1% tetracaine, the GERPs were tested again using the NaCl stimulus, and the aforementioned waveforms disappeared (Figure 4B).

### 3.3 Effects of stimulus concentration on latency and amplitude of GERPs

As the concentration of the salty stimulus increased, the latency of P1 and P2 at Fz and Cz significantly decreased (see Table 2, Figure 5). The paired *t*-test revealed no significant differences in the amplitudes of N1-P1 and N2-P2 at both Fz and Cz between the low and high concentrations of salty taste stimuli (Figure 5). Noticeably, some patients (48%) showed an increase in the amplitudes of P1-N1 or P2-N2 as the intensity of the saltiness stimulus increased, as depicted in Figure 4.

**Table 2:** Paired *t*-tests revealed comparisons of GERPs parameters (latency and amplitude) across different stimulation sites and various stimulation concentrations.

Paired *t* test	FZ-Cz				0.3–0.6			
Amplitude	0.3 N1-P1	0.3 N2-P2	0.6 N1-P1	0.6 N2-P2	Fz N1-P1	Fz N2-P2	Cz N1-P1	Cz N2-P2
*p* value	0.3	0.006	0.3	0.01	0.9	0.8	0.9	0.9
*t*	*t* = 1.1, df = 45	*t* = 2.9, df = 45	*t* = 1, df = 45	*t* = 2.6, df = 45	*t* = 0.9, df = 45	*t* = 0.3, df = 45	*t* = 0.1, df = 45	*t* = 0.2, df = 45
Latency	0.3P1	0.3P2	0.6P1	0.6P2	Fz P1	Fz P2	Cz P1	Cz P2
*p* value	0.5	0.5	0.2	0.8	0.001	0.002	0.003	0.009
*t*	*t* = 0.7, df = 45	*t* = 0.6, df = 45	*t* = 1.2, df = 45	*t* = 0.3, df = 45	*t* = 3.4, df = 45	*t* = 3.3, df = 45	*t* = 3.1, df = 45	*t* = 2.7, df = 45

### 3.4 Correlation between psychophysical gustatory test scores and GERPs

**Figure 6:**
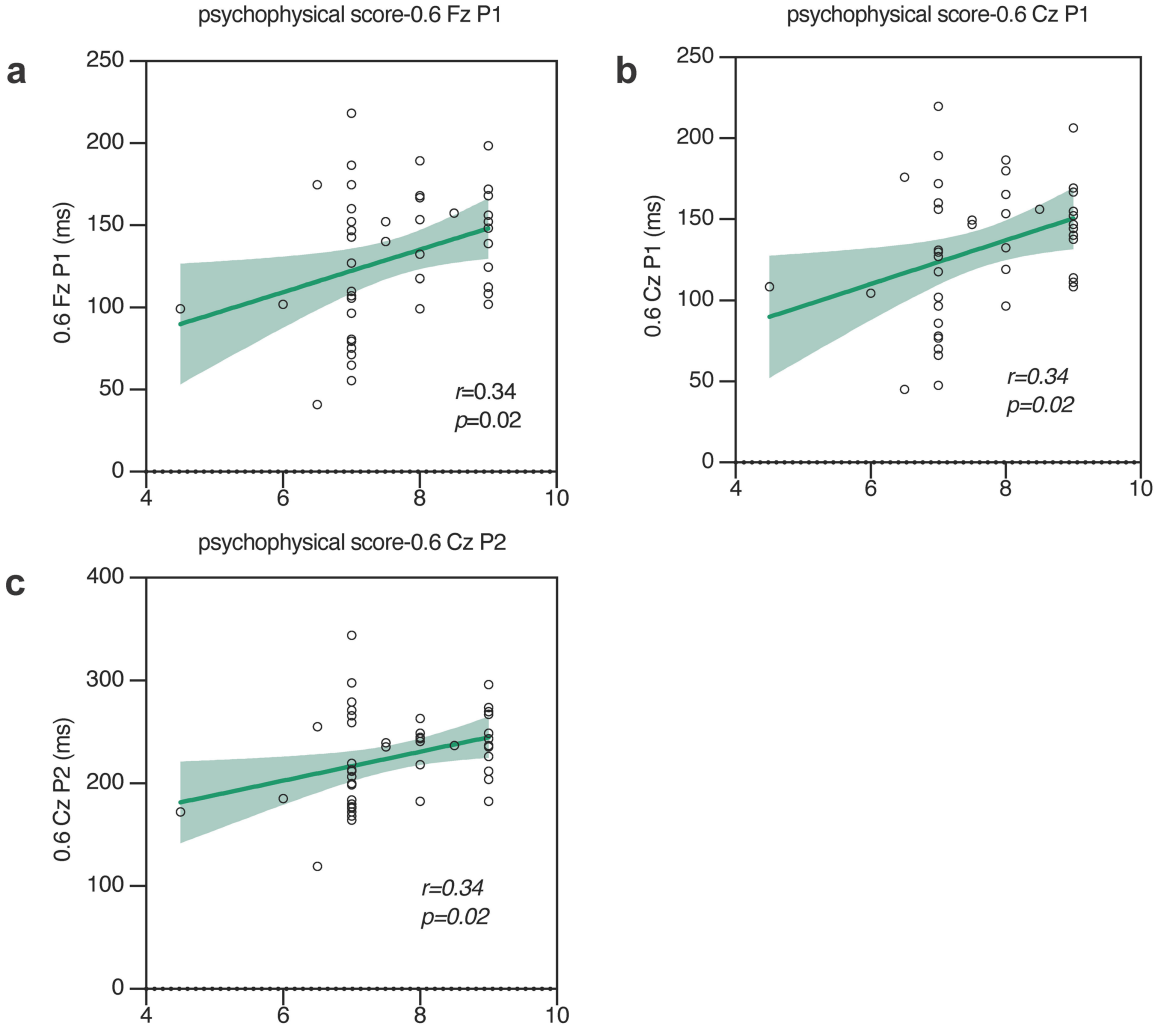
Analysis of the correlation between various GERP components and taste psychophysical tests. The latency of P1 **(a,b)** and P2 **(c)** are positively correlated with taste psychophysical tests. (F: Fz; C: Cz; 0.3: 0.3% NaCl; 0.6: 0.6% NaCl).

The psychophysical gustatory test scores of sucrose, NaCl, citric acid, and quinine hydrochloride were 8.3 ± 1.3, 7.7 ± 1, 8.8 ± 0.4, and 7.6 ± 0.9, respectively. The analysis of simple linear regression on the correlation between psychophysical gustatory function test and the parameters of GERP waveforms revealed that the psychophysical taste test scores for salt were positively correlated with the latency of P1 (Fz: *p* = 0.02, *r*^2^ = 0.1; Cz: *p* = 0.02, *r*^2^ = 0.1) at Fz and Cz and P2 (Cz: *p* = 0.02, *r*^2^ = 0.1) at Cz in response to 0.6% NaCl stimulation, as shown in Figure 6. This study did not find a correlation between other psychophysical taste test scores, namely sweet, sour, or bitter tastes, and the parameters of gustatory event-related potential peaks.

## 4 Discussion

A performance-stable and reliable gustometer has been developed by our team. This study reported an independently constructed gustometer and tested it in healthy young subjects. It successfully recorded stable and specific GERPs waveforms, capturing the classic peaks of GERPs (Hu et al., 2020). The flat curves with no peaks using distilled water as a control and the no-peak waveforms obtained after anesthetizing the tongue surface are consistent with previous findings (Kobal, 1985), which further validating the specificity of the potentials obtained. The major findings of the present preliminary study were that GERP (i) there was a concentration-specific topographical distribution indicating the activation of gustatory cortex changes as stimulus concentration, (ii) GERPs recorded at the Cz had higher amplitude and shorter latency for both P1 and P2 than recording postion Fz; (iii) the GERPs latencies of peaks exhibit a significant correlation with psychophysical gustatory test.

Gustometer development is key for obtaining reliable GERPs. To maximize the precision of stimulus delivery, the following issues need to be considered: the mechanical delay between trigger signal and the onset of gustatory stimulation, influenced by various variables such as the viscosity of the solution, the flow rate of the system, the length of the tubing, etc.; taste stimulus with as much of a square shape characteristic as possible; and eliminating influences of oral somatosensation, temperature, and oro-facial muscle movements. In recent decades, various taste stimulation methods have been explored to address these issues. The first GERPs to liquid stimuli were obtained almost 50 years ago. Funakoshi and Kawamura (1971) first attempted to describe cerebral taste event-related potentials via an apparatus consisting of a hinged spoon that delivered the taste solution (in a comparably large quantity) when tilted. Unfortunately, their research could not be replicated later by Schaupp (1971) or Bujas (1980). At the same time, the potential of electrical taste stimulation was explored, which applies electric pulses to lingual taste buds to elicit a unique taste percept with good stimulus control (Plattig, 1969), yet its ecological validity remains debatable. Recently, several mature gustometers have been reported by multiple centers. We have summarized and compared the origins, forms of gustatory stimulus delivery, latency of P1 and P2, amplitude of P1N1, and the appearance of these gustometers in Table 3. Agnès Jacquin-Piques from France, Burghart, Wedel from Germany, and Camilla Arndal Andersen from Denmark used an air fine spray to deliver tastants. The gustometer developed by Emerging Tech Trans LLC and our center used pumps to deliver liquid tastants.

**Table 3:** A brief comparison of the various gustometer design and GERPs data across different centers.

Region	Germany	Pennsylvania, USA	China	France	Denmark and Germany
Gustometer	Burghart, Wedel (Payne et al., 2024)	Emerging Tech Trans LLC (Nin and Tsuzuki, 2024)	Patent Granted	Jacquin-Piques A (Zhu et al., 2023; Iannilli et al., 2015)	Camilla Arndal Andersen Richard Höchenberger (Mouillot et al., 2019)
Stimulus form	Air stream with constant temperature MR-compatible	Liquid Pump drive Fully MR-compatible	Liquid Pump drive MR-compatible	Compressed air (controlled through a manometer).	Air spray MR-compatible
Principles and characteristics	Eliminate the influence of liquid temperature and tactile	Delivered solutions through peristaltic pumps and Teflon tubes. Each pump has a slightly different tone, which helps inform subjects which stimulus is currently active.	Two parallel silicone tubes were used: one from channel 4 for water and the other from bottles 1–3 for a taste solution	Two silicone tubes were used: one for the control solution, one for the taste solution. Electronic device controlled valve switching.	Computer-controlled, modular pump system
Need to swallow the stimulus solutions?	No	Yes	No	Yes, but small amount.	Yes, but small amount.
P1 latency(ms) at Cz	126.67 ± 31.95	131.82 ± 46.14 (0.3 M NaCl)	151.1 ± 34.9 (0.3% NaCl)	141.1 ± 21.0 (Sweet) 150 ± 17 (Sweet) 156 ± 30 (0.5% NaCl)	-
P2 latency(ms) at Cz	431.57 ± 44.19	414.12 ± 75.86 (0.3 M NaCl)	244.6 ± 37.3 (0.3% NaCl)	-	-
P1-N1 amplitude*/μV	5.91 ± 4.31	2.53 ± 1.89 (0.3 M NaCl)	5.1 ± 4.1 (0.3% NaCl)	24.8 ± 11.6 (Sweet) 19.7 ± 8.6 (0.5%NaCl)	-
P2-N2 amplitude/μV	10.65 ± 6.9	2.85 ± 2.42 (0.3 M NaCl)	4.9 ± 3.2 (0.3% NaCl)	-	-

*The amplitude of each response was calculated from positive to negative peaks.

The gustometer developed by our team represents some improvements. It allows participants to rest their chin on a stand while wearing headphones and an eye mask. These measures are designed to minimize the effects of sound and light, prevent electromyographic activity induced by swallowing, and eliminate the influence of tactile sensations and preconceived biases. Unlike the long testing durations typically used in earlier studies, which can negatively affect participants’ attention and task performance, our system incorporates a volume adjustment feature in the stimulator to regulate the amount of solution released. Through multiple trials, we identified the most comfortable stimulus volume for participants and adjusted the time interval between the salty and distilled water stimuli to generate a square-wave taste stimulus, characterized by a brief, rapid rise or fall. Reducing the sweep duration enhances synchronization of the electroencephalographic signals, resulting in cleaner and more consistent waveforms. Furthermore, compared to gustometers in other regions, our GERPs recording system does not require swallowing, which improves participant acceptance, allows for repeated testing, and reduces overall testing time (Table 3).

In this study, all subjects exhibited significant waveforms under salty stimuli. To further confirm that the elicited waveforms were taste-specific, we used water as a control, which did not produce any waveforms. After successfully eliciting salty GERPs, we anesthetized the facial and trigeminal nerve distribution areas on the tongue surface using tetracaine, which also failed to elicit GERPs. These findings confirm that the EEG signals we recorded were indeed induced by salty taste stimuli. Previous studies have found (Hu et al., 2020; Kobal, 1985; Tzieropoulos et al., 2013) that the average latency of the P1 is 70–150 ms, and the latency of the P2 is 350–500 ms. The average latency of the P2 induced by salty taste in this study was slightly shorter than the literature. Morphologically, the presently recorded GERP were similar to those described previously (Mizoguchi et al., 2002). Apart from this, all subjects showed significantly shortened latencies and partial exhibited greater amplitudes with increasing concentrations, which is in accordance with the recordings made by Kobal and Thomas Hummel, indicating that the increase in concentration modulated the amplitude and latency of the brain’s response (Hummel et al., 2010; Kobal, 1985; Tzieropoulos et al., 2013). However, we still observed that the amplitude decreased for other subjects as the stimuli increased. This inconsistency could be due to the short rest periods between the two concentration sections, a long recording session that may have resulted in desensitization (taste fatigue), or the possibility that the differences in concentration used at present were too small to elicit the differences that had been observed earlier using different setups and different stimulus concentrations (Singh et al., 2011), or individual differences in taste preferences. Therefore, further exploration of the factors affecting GERPs and the optimal stimulus concentration is needed.

Higher psychophysical gustatory test scores for salty taste correlated with higher responses in GERPs, further confirming the consistency of sensitivity between the peripheral gustatory system and the central nervous system. Although in this study there is a statistically significant linear correlation between the psychophysical test and the P1/P2 wave latency of the objective GERP, the maximum correlation coefficient (r-value) is only 0.34. The literature indicates that the psychophysical results of olfactory and gustatory functions do not linearly correlate with their event-related equivalents. This is why olfactory and gustatory event-related responses are currently only applicable for assessing the presence or absence of corresponding chemosensory functions (Ohla et al., 2012; Hummel et al., 2010; Lötsch and Hummel, 2006). This may be attributed to the fact that psychophysical tests themselves are influenced by various factors, such as the subject’s condition and outcome judgment, which lead to variability in results.

This study has some specific limitations that need addressing. Firstly, since NeuroAudio EEG is designed for auditory electrophysiological monitoring, it is equipped with only two EEG recording channels (Figure 2), which limits our ability to control for blinking artifacts with appropriate recordings like Fp2. Nonetheless, we mitigated eye movement and blinking contamination by using an eye mask and maintaining the subjects’ focus. Secondly, while the disappearance of GERPs following local anesthesia with tetracaine suggests specificity to gustatory input, it is important to acknowledge that local anesthesia only blocks peripheral nerve input and does not necessarily imply a complete absence of cortical processing. Future studies should include control ERP components, such as somatosensory or auditory ERPs, to ensure that the observed GERP disappearance is not due to a broader neural suppression effect. This approach will further validate the specificity of the recorded GERPs and strengthen the conclusions drawn from the study. Thirdly, this study validated the functionality of the gustometer by demonstrating that it reliably elicited GERPs in response to salty stimuli. While this represents an important first step, further validation across additional taste qualities is necessary to fully establish its clinical and research utility. Previous studies have reported GERPs for all five basic taste qualities, and future investigations will expand our testing to include sweet, sour, bitter, and umami stimuli. Last, several factors may contribute to variability in the recorded GERPs. Individual differences in gustatory sensitivity could have influenced response amplitudes, despite the use of psychophysical gustatory testing to confirm normal taste function. Additionally, repeated exposure to salty stimuli may have led to sensory adaptation, reducing responses over time. While inter-stimulus intervals were incorporated to minimize adaptation, future studies should consider randomized stimulus sequences to further control for this effect. The next step is to gradually advance the clinical transformation of the gustometer and GERPs device, launch corresponding commercial products, and then explore the value of GERPs in the diagnosis and treatment of taste disorder diseases.

In conclusion, the novel gustometer effectively records true and reliable GERP waveforms. This study validated the consistency of GERP amplitude and latency with psychophysical gustatory tests, highlighting the gustometer’s potential for clinical and research applications in gustatory system analysis.

## Data Availability

The datasets presented in this study can be found in online repositories. The names of the repository/repositories and accession number(s) can be found at: https://osf.io/7zpsy/?view_only=95382b60338e48d69e44b1b82c32d94e.
